# Scaffolded Medication Therapy Management in a Pharmacy Skills Laboratory: A Structured Approach to Skill Development

**DOI:** 10.3390/pharmacy13050132

**Published:** 2025-09-15

**Authors:** Kimberley J. Begley, Molly C. Goessling, Tara M. Eickhoff, Timothy P. Ivers

**Affiliations:** School of Pharmacy and Health Professions, Creighton University, 2500 California Plaza, Omaha, NE 68178, USA; mollygoessling@creighton.edu (M.C.G.); taraeickhoff@creighton.edu (T.M.E.);

**Keywords:** medication therapy management (MTM), pharmacy education, scaffolded instruction, clinical skill development

## Abstract

Pharmacists are increasingly expected to deliver medication therapy management (MTM) services, yet many pharmacy students report insufficient confidence and preparedness in executing these complex tasks. This study evaluated a scaffolded MTM instructional series integrated into a second-year pharmacy skills laboratory, aiming to enhance student competence through progressive, structured learning. A mixed-methods design assessed changes in self-reported confidence, performance-based outcomes, and reflective insights among 154 students across three educational tracks. The 14-week intervention included sequential activities such as medication history interviews, drug-related problem (DRP) identification, care plan development, and comprehensive MTM simulations. Pre- and post-intervention surveys revealed statistically significant improvements in all 18 confidence domains, with the greatest gains in therapeutic recommendations and prescriber communication. Effect sizes ranged from small to very large (Cohen’s d 0.33–1.05), indicating gains that were both statistically reliable and educationally meaningful. Performance assessments showed consistent proficiency across MTM components, with average scores ranging from 90% to 96%. Qualitative reflections reinforced these findings, highlighting growth in communication, individualized patient care, and professional identity formation. The scaffolded approach aligns with accreditation standards and instructional design theory, offering a model for pharmacy curricula. Despite limitations such as lack of a comparator group and potential response bias, the study demonstrates that scaffolded MTM instruction effectively supports skill acquisition and confidence, preparing students for real-world clinical practice.

## 1. Introduction

Medication therapy management (MTM) represents a vital component of contemporary pharmacy practice, as pharmacists play an increasingly integral role in optimizing therapeutic outcomes, reducing drug-related problems (DRPs), and contributing to cost-effective healthcare delivery [1,2,3]. MTM encompasses a spectrum of services including comprehensive medication reviews, patient education, therapeutic monitoring, and interprofessional collaboration. The ability to proficiently execute MTM services is fundamental to the practice-ready pharmacist envisioned by professional standards and accreditation bodies.

The Accreditation Council for Pharmacy Education (ACPE) Standards 2025 emphasize the need for pharmacy curricula to foster the development of students capable of delivering patient-centered care and engaging in collaborative practice. Within Standard 2 (Curriculum), the integration of clinical decision-making, therapeutic planning, and comprehensive medication management, key components of the Pharmacists’ Patient Care Process, is explicitly highlighted [4,5]. Similarly, the Center for the Advancement of Pharmacy Education (CAPE) 2013 educational outcomes align with Medication Therapy Management objectives within Domains 2 (Essentials for Practice and Care), 3 (Approach to Practice and Care), and 4 (Personal and Professional Development) [6]. These competencies are directly reflected in the COEPA’s numbered Entrustable Professional Activities for new pharmacy graduates, such as EPA 1 (“Collect information necessary to identify a patient’s medication-related problems and health-related needs”), EPA 2 (“Assess collected information to determine a patient’s medication-related problems and health-related needs”), EPA 3 (“Create a care plan in collaboration with the patient, others trusted by the patient, and other health professionals to optimize pharmacologic and nonpharmacologic treatment”), and EPA 6 (“Implement a care plan in collaboration with the patient, others trusted by the patient, and other health professionals”) [7].

Despite these strong frameworks, many pharmacy students report a lack of confidence and preparedness in delivering MTM services upon graduation [8,9,10,11,12]. This gap is often attributed to fragmented teaching approaches that focus more on theory than practice and that present MTM as a uniform skill rather than a complex, multifaceted process. To address this, scaffolding offers a practical pedagogical solution. Rooted in cognitive load theory and the four-component instructional design model, scaffolding involves breaking down complex tasks into smaller, more manageable units of learning that gradually build toward independent mastery [13,14,15].

Scaffolding is widely used in health professions education to address the layered nature of clinical reasoning, communication, and problem-solving. In medical education, structured scaffolding has been shown to improve diagnostic accuracy and decision-making by offering learners guided practice with feedback before progressing to independent tasks [16]. In nursing education, scaffolded simulations improved students’ confidence in clinical reasoning, judgment, and critical thinking while helping them better manage evolving patient situations [17]. Studies have also shown that scaffolding promotes metacognition and encourages reflective practice, key elements of lifelong learning in healthcare [18,19]. Within interprofessional education, scaffolding supports role clarity and coordinated team-based care by allowing learners to build confidence within their professional scope before collaborating across disciplines [20].

In pharmacy education, scaffolding in laboratory and simulation-based settings has improved skill acquisition and performance in complex patient care scenarios [21,22]. Within the context of MTM, scaffolding may provide a framework through which students can sequentially acquire, integrate, and apply skills such as medication history taking, problem identification, clinical prioritization, and therapeutic planning. When implemented in a skills-based laboratory setting, scaffolded MTM activities can replicate authentic practice scenarios while providing a supportive environment for deliberate practice and feedback.

This study evaluates the implementation of a scaffolded MTM learning series integrated into a second-year pharmacy skills laboratory. Specifically, it investigates the impact of this approach on students’ confidence and competence in delivering MTM services, with a focus on breaking the MTM process into digestible, teachable elements that align with professional expectations.

## 2. Methods

### 2.1. Study Design and Objectives

A mixed-methods study was conducted to assess the effectiveness of scaffolded MTM instruction on student learning outcomes. The primary objective was to evaluate changes in student self-reported confidence and perceived competence in core MTM domains. Secondary objectives included assessing skill acquisition and integration through performance-based assessments and reflective analyses.

### 2.2. Participants

The study population included 154 second-year (P2) pharmacy students enrolled in a required skills laboratory course (PH A 469: Dispensing and Patient Care II) delivered across three educational tracks, namely the Omaha campus, Phoenix campus, and a distance-based cohort in the United States. All cohorts participated in a uniform curriculum and assessment schedule. The Creighton University PharmD program is a four-year curriculum, with three years of didactic instruction followed by a fourth year of advanced pharmacy practice experiences (APPEs). The P2 students in this study were enrolled during the spring semester, when the Dispensing and Patient Care II laboratory course aligns with concurrent pharmacotherapeutics coursework. This was the first point in the curriculum where students received structured MTM training. While formal demographics such as age and GPA were not collected, the class included approximately 82% female and 18% male students across the three educational pathways.

### 2.3. Scaffolded MTM Intervention

Faculty collaborated with clinical MTM practitioners to develop and implement a 14-week scaffolded instructional series. The series was designed to align with MTM core elements outlined by the American Pharmacists Association and other guiding bodies. All students across Omaha, Phoenix, and distance pathways completed the laboratory activities in person. The instructional design emphasized active, experiential learning and reproducibility: faculty portrayed patients during medication history interviews; case-based discussions were facilitated in small groups; and guided worksheets were used to structure DRP identification and prioritization. Summative assessments were organized in an OSCE-style format to mirror authentic MTM encounters while isolating distinct components for mastery. Learning activities were supported by standardized rubrics, structured faculty feedback, and peer discussion to promote consistency and reproducibility. The intervention utilized an intentional progression of complexity across multiple learning activities:

Weeks 1–2: Foundational lecture on MTM, core elements, and types of DRPs.
Weeks 3–5: Medication history interviews and prioritization of DRPs.
Weeks 6–8: Guided DRP identification through faculty-led case studies with structured worksheets.
Weeks 9–10: Case-based care plan development including prioritization and written provider and patient communication.
Weeks 11–14: Comprehensive MTM simulation requiring assessment, therapeutic recommendations, and written documentation.

Each activity was designed to scaffold student learning by breaking down complex MTM skills into progressive, manageable steps that built toward independent performance in the final simulation.

### 2.4. Data Collection and Analysis

Students completed an 18-item pre- and post-intervention survey using a 5-point Likert scale assessing confidence in areas such as information gathering, DRP identification, problem prioritization, patient communication, and plan development. No validated survey was identified in the literature; therefore, the authors developed the survey items to align with MTM competencies. The instrument was pilot-tested with faculty to ensure clarity and alignment with intended outcomes, supporting face and content validity; however, formal factor analysis was not conducted. The complete 18-item survey is provided in Supplementary Material File S1. Survey data were analyzed using paired *t*-tests. Analyses were conducted using SPSS v29 (IBM Corp. Released 2022. IBM SPSS Statistics for Windows, Version 29.0. IBM Corp., Armonk, NY, USA), with paired-sample *t*-tests applied to normally distributed differences and a significance threshold set to *p* < 0.05. Assumptions for the paired-sample *t*-test were verified, and the distribution of difference scores did not deviate substantially from normality, supporting the appropriateness of this analytic approach. Cohen’s d effect sizes were calculated to quantify the magnitude of pre–post differences. Performance data were extracted from the learning management system and analyzed for trends (including scores from medication history activities, DRP identification and classification exercises, written communication to providers, and overall MTM case performance). Student reflections were examined qualitatively using inductive thematic coding. Responses were coded thematically using conventional content analysis as described by Hsieh and Shannon [23]. Two independent coders reviewed the reflective narratives, identified key themes, and resolved discrepancies through discussion to ensure consistency and trustworthiness of the findings.

## 3. Results

### 3.1. Survey and Confidence Outcomes

A total of 126 students completed both the pre- and post-intervention surveys and were included in the paired analysis, yielding a response rate of 81%. Statistically significant improvements (*p* < 0.05) were observed across all 18 domains. The largest gains were noted in students’ perceived ability to recommend pharmacologic therapies for DRPs (mean increase = 0.69, *p* < 0.001), communicate DRP recommendations to prescribers (mean increase = 0.60, *p* < 0.001), and recognize the social determinants of health (mean increase = 0.48, *p* < 0.001) (Table 1). All *p*-values remained statistically significant with the larger sample (n = 126), all remained at *p* < 0.001 across the 18 survey domains. These results were both statistically significant and educationally meaningful. The greatest effect sizes were observed in domains related to recommending pharmacologic therapies (d = 1.05), communicating recommendations to prescribers (d = 0.74), and recognizing the social determinants of health (d = 0.67), demonstrating that the scaffolded instructional design had its most substantial impact on clinical decision-making and communication skills.

### 3.2. Performance-Based Assessments

Student performance demonstrated progressive improvement across the scaffolded MTM instructional series and is summarized in Table 2. In the medication history activity, students achieved an average score of 90% (SD = 3.8; range 82–98), reflecting strong foundational skills in patient interviewing and data collection. This was followed by the clinical prioritization case, where students excelled with an average score of 95% (SD = 4.9; range 85–100), demonstrating their ability to assess and rank DRPs effectively. In two subsequent complex cases focused on DRP identification and resolution, students maintained high proficiency with average scores of 92% (SD = 2.6; range 87–97) and 96% (SD = 4.8; range 86–100), respectively. The final MTM simulation, which incorporated embedded challenges and faculty portraying patients, required students to synthesize all prior learning to identify and resolve DRPs. Students performed well in this capstone activity, achieving an average score of 90% (SD = 4.1; range 82–98), demonstrating their readiness to apply MTM principles in realistic clinical scenarios.

### 3.3. Academic Performance and Grades

Overall course performance mirrored trends seen in MTM-specific assessments. The majority of students achieved A or B grades across the MTM modules, indicating consistent competence. Faculty rubric reviews confirmed students met expected performance thresholds on case documentation, therapeutic recommendations, and problem prioritization.

### 3.4. Qualitative Analysis of Reflections

A thematic analysis of student reflections on MTM activities revealed several key themes central to the development of professional competence. Representative student quotes for each theme are provided in Table 3.

Communication and empathy emerged as foundational skills. Students emphasized the importance of asking targeted questions, building rapport, and addressing sensitive topics such as adherence in a supportive, nonjudgmental manner: “You don’t want to be shaming them if they miss doses but instead you can identify why they are missing doses if you go about it nicely and show that you are on their side.”

Students also demonstrated a growing appreciation for the MTM process and comprehensive assessment, recognizing the value of obtaining complete medication histories, identifying therapy duplications and drug interactions, and considering contextual factors that influence care: “It is important to get all of the pertinent information on each drug that is taken to be able to fully assess any therapy duplications, adherence concerns, drug–drug interactions, drug-disease interactions, etc.” Structured tools such as SCHOLAR-MAC were noted as helpful frameworks for promoting systematic assessment.

Another recurring theme was the need for individualized and holistic care. Students reflected on the importance of understanding patient priorities and “looking at the whole patient rather than just a single disease state.” This included recognition that social determinants of health (SDOH), such as financial barriers, transportation, and health literacy, significantly influence outcomes: “It’s important to understand what might be preventing a patient from taking their medications as prescribed, whether it’s cost, transportation, a cultural barrier, or something else.”

Finally, students highlighted their role in patient education and empowerment, noting that many patients lack awareness of their medications and conditions: “I learned that patients often don’t realize what they’re taking and for what reason.”

Reflections also underscored the need for ongoing practice to build proficiency: “I would like to have more opportunities to practice the skill during the semester so that I can be more prepared.”

Collectively, these reflections illustrate how scaffolded MTM instruction supported growth in communication, assessment, individualized care, and patient empowerment, aligning with the broader competencies expected of pharmacists.

## 4. Discussion

These findings support prior reports that scaffolded instruction improves competence and confidence in complex clinical skills. In pharmacy education, scaffolded instruction has been successfully used to support clinical reasoning, therapeutic decision-making, and communication skills in simulated settings [24,25]. Although mean changes were modest (0.3–0.7 points), effect sizes (Cohen’s d 0.33–1.05) demonstrated meaningful improvements that were both statistically and educationally significant. Gains were particularly strong in pharmacotherapy recommendations and prescriber communication, reinforcing the impact of scaffolded instruction on critical domains of MTM competence.

Students in this study demonstrated strong longitudinal performance across the scaffolded MTM series. The use of progressive complexity—from medication history and prioritization to therapeutic planning—allowed learners to develop foundational confidence before integrating higher-order clinical tasks. This scaffolding approach applies principles from cognitive load theory, including reducing extraneous demands and supporting progressive skill integration through the four-component instructional design model (4C-ID) [13,26,27].

Although performance scores were high, this likely reflects the intentional scaffolding of difficulty rather than overly simplified cases. Early activities emphasized foundational skills such as medication histories, while subsequent cases required increasing levels of integration, culminating in a comprehensive MTM simulation with embedded challenges. All cases were reviewed by MTM faculty for rigor, and faculty-designed standardized rubrics were used to ensure consistent expectations across assessments. Thus, the consistently strong performance across activities can be interpreted as evidence of progressive skill development supported by structured scaffolding, rather than a function of case simplicity.

The statistically significant gains in DRP prioritization and duplicate therapy identification align with CAPE and EPA expectations for pharmacy graduates. Reflective narratives reinforced that students appreciated the sequencing of skills and the embedded nature of complexity in simulated patients. These experiences likely fostered metacognition and professional identity formation [28].

Similar findings have been reported in other pharmacy and health profession education contexts, where scaffolded case-based or skill-based learning improved students’ confidence and competence in MTM or related domains. For example, Begley et al. [29] reported that pharmacy students demonstrated significant improvements in MTM documentation and case resolution skills after progressive case-based training, while Adrian [30] found that structured role-play enhanced confidence pharmacy students’ oral communication skills and self-perceived competence in patient care communication. Our results are consistent with these studies, and the magnitude of improvement observed in our model, particularly in areas such as pharmacotherapy recommendations and prescriber communication, suggests that scaffolded MTM instruction can demonstrate meaningful improvements even in early stages of the curriculum.

Because students had not previously received formal MTM training, it is reasonable that self-assessed competence improved markedly across all domains. We acknowledge that self-assessment may logically favor the intervention, as students were exposed to new content for the first time; however, these gains were corroborated by high performance in structured, performance-based assessments, strengthening the validity of the findings. Triangulation supports the interpretation that scaffolded MTM instruction supported both perceived and demonstrated competence.

## 5. Limitations

While outcomes were favorable, this study has several limitations. First, the lack of a comparator group prevents a definitive attribution of improved performance solely to the scaffolding intervention. Second, student-reported confidence may not equate directly to clinical readiness, although previous research indicates that scaffolding can support both self-efficacy and objective performance. Additionally, self-reported survey data may be subject to response bias, potentially inflating perceived gains. Although a standardized rubric was used for grading, multiple faculty members were involved in evaluating student performance, which may have introduced variability despite calibration efforts. Finally, this was a single-institution study, which may limit generalizability, and implementation required additional faculty resources for patient role-play and individualized assessment.

## 6. Conclusions

Integrating scaffolded MTM instruction within a pharmacy skills laboratory improved student confidence and demonstrated competence across key MTM domains. The stepwise design allowed for focused learning, regular feedback, and a progressive integration of skills. This approach aligns with accreditation standards, CAPE outcomes, and EPAs, and it offers a replicable model for enhancing MTM instruction in pharmacy curricula.

## Figures and Tables

**Table 1:** Medication therapy management survey paired *t*-test results with Cohen’s d (n = 126).

Survey Question	Pre (Mean ± SD)	Post (Mean ± SD)	*p*-Value	Cohen’s D
Confident in obtaining a complete medication history	4.03 (0.75)	4.43 (0.53)	2.150 × 10^−10^	0.616
Confident in identifying a patient’s chief complaint	4.52 (0.71)	4.72 (0.48)	3.166 × 10^−4^	0.330
Confident in gathering and applying HPI details	4.17 (0.91)	4.57 (0.50)	1.138 × 10^−8^	0.545
Confident in applying patient data to care decisions	3.91 (0.82)	4.32 (0.59)	2.298 × 10^−9^	0.574
Confident in identifying and addressing medication nonadherence	4.23 (0.72)	4.62 (0.52)	1.611 × 10^−10^	0.621
Confident in recognizing social determinants of health	3.97 (0.85)	4.45 (0.56)	1.112 × 10^−11^	0.667
Confident in identifying drug-related problems (DRPs)	3.71 (0.80)	4.18 (0.66)	5.101 × 10^−11^	0.641
Confident in prioritizing drug-related problems	3.78 (0.89)	4.20 (0.81)	1.706 × 10^−7^	0.494
Confident in categorizing drug-related problems	3.87 (0.89)	4.34 (0.69)	9.266 × 10^−10^	0.590
Confident in assessing patient issues using available information	4.01 (0.86)	4.40 (0.63)	4.938 × 10^−8^	0.517
Confident in recommending pharmacologic therapies for DRPs	3.60 (0.75)	4.29 (0.55)	5.326 × 10^−22^	1.049
Confident in recommending non-pharmacologic therapies for DRPs	3.91 (0.82)	4.37 (0.67)	2.360 × 10^−10^	0.614
Confident in communicating DRP recommendations to prescribers	3.80 (0.94)	4.40 (0.66)	1.490 × 10^−13^	0.739
Confident in counseling patients on DRPs and actionable steps	4.05 (0.82)	4.43 (0.56)	1.383 × 10^−8^	0.541
Believe MTM improves patient medication outcomes	4.46 (0.73)	4.72 (0.45)	4.208 × 10^−6^	0.429
Believe pharmacist-led MTM advances the profession	4.60 (0.70)	4.80 (0.40)	1.357 × 10^−4^	0.351
Believe MTM can reduce healthcare costs	4.40 (0.84)	4.68 (0.53)	1.697 × 10^−5^	0.399
Believe learning MTM in segments enhances understanding	4.49 (0.73)	4.71 (0.55)	2.084 × 10^−4^	0.340

All comparisons are statistically significant at *p* < 0.001.

**Table 2:** Student performance across scaffolded MTM activities (n = 126).

Activity	Average Score (%)	SD	Range (%)
Medication History	90	3.8	82–98
Clinical Prioritization	95	4.9	85–100
DRP Case 1	92	2.6	87–97
DRP Case 2	96	4.8	86–100
Final MTM Simulation	90	4.1	82–98

**Table 3:** Student representative quotes from reflections on scaffolded MTM activities.

Theme	Representative Student Quotes
Communication and Empathy	1. “You don’t want to be shaming them if they miss doses but instead you can identify why they are missing doses if you go about it nicely and show that you are on their side.” 2. “I learned how to correctly ask sensitive questions to the patient and obtain all of the information and detail I need to make appropriate recommendations to my patient.” 3. “Establishing trust with the patient is key because they are more willing to share important details when they feel supported.”
Comprehensive Assessment	1. “It is important to get all of the pertinent information on each drug that is taken to be able to fully assess any therapy duplications, adherence concerns, drug-drug interactions, drug-disease interactions, etc.” 2. “Gathering all components of SCHOLAR-MAC helped me systematically collect patient information and make an appropriate recommendation.” 3. “I learned that every detail matters—sometimes even small lifestyle habits can change how I evaluate medications and problems.”
Individualized and Holistic Care	1. “It’s important to understand what might be preventing a patient from taking their medications as prescribed, whether it’s cost, transportation, a cultural barrier, or something else.” 2. “Looking at the whole patient rather than just a single disease state.” 3. “Patients will have different priorities with problems than myself and you sometimes have to dig for the answers.”
Patient Empowerment	1. “I learned that patients often don’t realize what they’re taking and for what reason.” 2. “I realized patients need help understanding their medications in simple terms, otherwise they can’t take control of their health.” 3. “Educating patients about their medications helps them feel more confident and engaged in their care.”
Continuous Learning and Practice	1. “I would like to have more opportunities to practice the skill during the semester so that I can be more prepared.” 2. “I learned that MTM requires constant practice and feedback to feel comfortable.” 3. “Each time I practiced MTM I gained a little more confidence, showing me the importance of repetition in skill development.”

## Data Availability

The data presented in this study are available on request from the corresponding author. The data are not publicly available due to restrictions related to student privacy and confidentiality.

## Supplementary Materials

The following supporting information can be downloaded at: https://www.mdpi.com/article/10.3390/pharmacy13050132/s1, File S1: Survey Questions.

## Abbreviations

The following abbreviations are used in this manuscript:

MTM	Medication therapy management
DRP	Drug-related problem
SD	Standard deviation
SCHOLAR-MAC	Symptoms, characteristics, history, onset, location, aggravating factors, remitting factors, medications, allergies, and conditions
