# Supplementary material for: Scaffolded Medication Therapy Management in a Pharmacy Skills Laboratory: A Structured Approach to Skill Development
Source: Pharmacy (Basel). 2025 Sep 15;13(5):132. doi: 10.3390/pharmacy13050132 (PMC12452581; doi:10.3390/pharmacy13050132)
Supplement: Supplementary file 1 [file pharmacy-13-00132-s001.zip › pharmacy-3836173-supplementary.pdf]

File S1. Survey Questions

Likert Scale:

1 = Strongly disagree / Not confident at all
2 = Disagree / Slightly confident
3 = Neither agree nor disagree / Moderately confident
4 = Agree / Confident
5 = Strongly agree / Very confident

1. How confident are you in your ability to obtain a complete medication history?

2. How confident are you in your ability to identify a patient's chief complaint?

3. How confident are you in your ability to gather and apply details of the history of present illness (e.g., signs and symptoms, duration, aggravating and remitting factors)?

4. How confident are you in your ability to apply patient data (e.g., vitals, labs, risk assessments) to care decisions?

5. How confident are you in your ability to identify and address medication nonadherence?

6. How confident are you in your ability to recognize social determinants of health that may affect a patient?

7. How confident are you in your ability to identify drug-related problems (DRPs)?

8. How confident are you in your ability to prioritize drug-related problems (DRPs)?

9. How confident are you in your ability to categorize drug-related problems (DRPs)?

10. How confident are you in your ability to assess patient issues using available information?

11. How confident are you in your ability to recommend a pharmacologic therapy to address a drug-related problem?

12. How confident are you in your ability to recommend a nonpharmacologic therapy to address a drug-related problem?

13. How confident are you in your ability to communicate your recommendations about drug-related problems to prescribers?

14. How confident are you in your ability to counsel patients about drug-related problems and provide actionable steps?

15. To what extent do you agree that providing medication therapy management (MTM) improves patient medication outcomes?

16. To what extent do you agree that pharmacist participation in MTM is an important step in advancing the profession?

17. To what extent do you agree that providing MTM services can reduce healthcare costs?

18. To what extent do you agree that learning MTM in segments enhances understanding and ability to apply these skills?
